# SerpinB2 deficiency is associated with delayed mammary tumor development and decreased pro-tumorigenic macrophage polarization

**DOI:** 10.1186/s12885-024-12473-6

**Published:** 2024-07-03

**Authors:** Yin Ji Piao, Hoe Suk Kim, Hyelim Kim, Jun Shen, Woo Kyung Moon

**Affiliations:** 1 Department of Radiology, Sun Yat-Sen Memorial Hospital and Sun Yat-Sen University, No. 107 Yanjiang Road West, Guangzhou, 510120 China; 2 Department of Biomedical Sciences, Seoul National University College of Medicine, 103 Daehak-Ro, Jongno-Gu, Seoul, 03080 Korea; 3 Department of Radiology, Seoul National University Hospital and Seoul National University College of Medicine, 101 Daehak-Ro, Jongno-Gu, Seoul, 03080 Korea; 4 Biomedical Research Institute, Seoul National University Hospital, 101 Daehak-Ro, Jongno-Gu, Seoul, 03080 Korea; 5 Integrated Major in Innovative Medical Science, Seoul National University Graduate School, 103 Daehak-Ro, Jongno-Gu, Seoul, 03080 Korea; 6 Department of Pharmaceutical Sciences, College of Pharmacy & Allied Health Professions, South Dakota State University, SAV# 255, Box 2202C, Brookings, SD 57007 USA

**Keywords:** SerpinB2, Breast cancer, MMTV-PyMT transgenic mouse, Tumor-associated macrophage

## Abstract

The in vivo functions of SerpinB2 in tumor cells and tumor-associated macrophages (TAMs) during breast cancer development and metastasis remain elusive. SerpinB2-deficient MMTV-PyMT mice (PyMT^SB2−/−^) were previously produced to explore the biological roles of SerpinB2 in breast cancer. Compared with MMTV-PyMT wild-type (PyMT^WT^) mice, PyMT^SB2−/−^ mice showed delayed tumor progression and reduced CK8 + tumor cell dissemination to lymph nodes. RNA-Seq data revealed significantly enriched genes associated with inflammatory responses, especially upregulated M1 and downregulated M2 macrophage marker genes in PyMT^SB2−/−^ tumors. Decreased CD206^+^M2 and increased NOS2^+^M1 markers were detected in the primary tumors and metastatic lymph nodes of PyMT^SB2−/−^ mice. In an in vitro study, SerpinB2 knockdown decreased the sphere formation and migration of MDA-MB-231 cells and suppressed protumorigenic M2 polarization of RAW264.7 cells. The combination of low SerpinB2, high NOS2, and low CD206 expression was favorable for survival in patients with breast cancer, as assessed in the BreastMark dataset. Our study demonstrates that SerpinB2 deficiency delays mammary tumor development and metastasis in PyMT^WT^ mice, along with reduced sphere formation and migration abilities of tumor cells and decreased macrophage protumorigenic polarization.

**Supplementary Information:**

The online version contains supplementary material available at 10.1186/s12885-024-12473-6.

## Introduction

SerpinB2, a plasminogen activator inhibitor-2 (PAI-2), is a serine protease inhibitor of the serpin superfamily that inactivates tissue plasminogen activator (tPA) and urokinase plasminogen activator (uPA) [1]. The role of SerpinB2 in breast cancer growth and metastasis is complex and controversial because SerpinB2 may have both pro-tumor and anti-tumor effects [2]. High expression of tumor cell-associated SerpinB2 facilitates breast cancer cell survival and metastasis by protecting breast cancer cells from death signals, promoting breast cancer cell migration ability, and enhancing macrophage recruitment into tumor tissues [3, 4]. In contrast, SerpinB2 expression by breast cancer cells significantly inhibits metastasis by inhibiting extracellular uPA [5, 6]. There is a significant association between SerpinB2 level and survival; breast cancer cell-associated SerpinB2 is identified as an unfavorable prognostic indicator [3, 4]. Nevertheless, the precise function of SerpinB2 in vivo regarding breast cancer progression and metastasis remains uncertain.

Among the immune cells recruited to the breast tumor, macrophages are one of the most abundant immune cell types shown to play a role as a facilitator for breast tumor development and progression at all stages [7, 8]. In macrophages, SerpinB2 is strongly upregulated by many inflammatory and/or stress-related factors and is involved in macrophage immune function regulation [9, 10]. SerpinB2 has also been implicated in the tumor suppression and promotion functions of TAMs [5]. TAMs undergoing different classic (M1) or alternative (M2) phenotypic polarization express the specific M1 marker nitric oxide synthase-2 (NOS2) or the M2 markers CD206 which are believed to hold tumor-preventing or -promoting activities [11]. An increase in SerpinB2 protein levels is observed in both M1 and M2 macrophages; notably, SerpinB2 protein levels are higher in M2 than in M1 macrophages [12]. SerpinB2 has been reported to belong to the pro-tumorigenic M2-associated gene family [9, 13–15]. The role of SerpinB2 in controlling TAM immune responses in mammary cancer development and progression has not been well studied.

We previously observed that SerpinB2 deficiency changes the expression of numerous genes, specifically those involved in the immunological and inflammatory response found in mammary tumors of PyMT^SB2−/−^ mice [16]. In this study, we explored different TAM polarizations as well as mammary cancer progression and metastasis in PyMT^SB2−/−^ mice. Our study indicated a substantial postponement in the development of primary tumors and the metastasis to lymph nodes (LN) in PyMT^SB2−/−^ mice, accompanied by decreased pro-tumorigenic polarization of TAMs that exhibited low CD206 and high NOS2 expression. Additionally, an in vitro study revealed that knocking down SerpinB2 in MDA-MB-231 breast cancer cells and RAW264.7 macrophages decreased breast cancer cell migration and sphere formation and the pro-tumorigenic polarization of macrophages. We verified the clinical significance of the combination of low SerpinB2, high NOS2, and low CD206 expression as predictive indicators for good patient survival using the online web-based service BreastMark.

## Materials and methods

### Cell cultures

MDA-MB-231 cells and RAW264.7 cells (Korean Cell Line Bank, Seoul, Korea) were cultured in RPMI-1640 (WelGENE, Seoul, Korea) and were grown in a 5% CO_2_ incubator at 37 °C. Lentiviral vectors encoding SerpinB2, including short hairpin RNA (shRNA) targeting SerpinB2 and scrambled shRNA, were purchased from Dharmacon (Lafayette, CO, USA). SerpinB2-overexpressing and -knockdown MDA-MB-231 cells were produced by the transduction of the SerpinB2 gene or shRNA using lentivirus [4]. SerpinB2 siRNAs were purchased from Santa Cruz Biotechnology (Dallas, TXs, USA). RAW264.7 cells were transfected with siRNAs using Lipofectamine-2000 (Invitrogen, Carlsbad, CA, USA).

### Antibodies

The following primary antibodies were used for detecting β-actin, SerpinB2, SerpinE1, uPA, CD206 (Abcam, Cambridge, MA, USA), NOS2 (Santa Cruz Biotechnology), F4/80 (Invitrogen, Carlsbad, CA, USA), cytokeratin-8/18 (CK8) (Developmental Studies Hybridoma Bank, Iowa City, IA,USA) by performing western blot, immunohistochemistry, and immunofluorescence staining. Horseradish peroxidase-conjugated secondary antibodies and Alexa Fluor 488 or 594 -conjugated secondary antibodies (Thermo Fisher Scientific, Waltham, MA, USA) were used.

### Animals

The MMTV-PyMT mice on the C57BL/6 background, initially created by Muller's research group [17], were generously supplied by Dr. Sandra Gendler (Mayo Clinic in Scottsdale, AZ). The SerpinB2-deficient (B6.129S1-Serpinb2tm1Dgi/J) (SB2 − / −) mice in the C57BL/6 background were obtained from the Jackson Laboratory (Bar Harbor, ME) for this study. PyMT^SB2−/−^ mice were generated by mating male PyMT^WT^ mice with female SB2 − / − mice, both in the C57BL/6 background. The genotyping of PyMT^WT^ and PyMT^SB2−/−^ mice was conducted using methods previously described [16]. Zoletil (5 mg/kg of mouse body weight) was administrated intramuscularly to excise mammary tumor tissues and axillary lymph nodes from 16- 25-week-old PyMT^WT^ and PyMT^SB2−/−^ mice. Subsequently, the mice were transferred to a CO_2_ chamber for euthanasia, where CO_2_ was gradually introduced to fill 30–70% of the chamber volume per minute. The mice were continuously exposed to the flowing CO_2_ for > 1 min following the cessation of breathing to ensure death. The Biomedical Center for Animal Resource Development of Seoul National University (SNU) provided animal care for all mice. All animal care and experimental procedures were conducted according to the National Research Council's guideline approved by the SNU Institutional Animal Care and Use Committee (SNU-150210–3-4). The experimental methods described in this study follow the ARRIVE guidelines (available at https://arriveguidelines.org) for reporting.

### RNA-sequencing and differential gene functional annotation

For the study of RNA-sequencing (RNA-Seq), tumor tissues were obtained from 8 female mice of PyMT^WT^ and PyMT^SB2−/−^ groups (20 weeks old [*n* = 1], 22 weeks old [*n* = 2], and 25 weeks old [*n* = 5]), because tumor tissues obtained from mice at 16 weeks of age were found to have limited sizes. To visualize the results of differential gene expression (DEGs) between PyMT^WT^ and PyMT^SB2−/−^ tumors, a volcano plot and heat map were created using EdgeR within R (R development Core Team, 2016) through Bioconductor [18]. Genes with a p-value below 0.05 and an absolute log2-fold change (FC) exceeding 1.5 were deemed significant. Furthermore, significant Gene Ontology (GO) terms associated with the DEGs were identified. The Benjamini–Hochberg method was employed for multiple testing adjustments, with a threshold of Benjamini < 0.05 considered statistically significant. The resulting GO term lists, specifically focusing on the biological process (BP) category, were compared and matched with each other.

### Quantitative real‑time RT‑PCR

To verify the expression levels of genes identified from RNA-Seq, quantitative real-time RT-PCR (qRT-PCR) was performed using the same samples (*n* = 8) which used for RNA-Seq and specific primer sets for (Table S1). Lipopolysaccharides (LPS, 100 ng/ml, Sigma-Aldrich, St. Louis, MO, USA) was used to induce the upregulation of SerpinB2 in RAW264.7 cells as a positive control. The results were analyzed using the 2^–∆∆Ct^ method.

### Western blot 

For western blots, the protein lysates were extracted from the tumor tissues of PyMT^WT^ and PyMT^SB2−/−^ female mice (25-week-old). The cells and tumor tissues were lysed in RIPA buffer (Sigma-Adrich). The intensity of each band was quantified by ImageJ software (NIH, Bethesda, MD, USA). Relative protein levels were expressed after normalizing their intensities with the intensity of β-actin. The Western blot's raw data is presented in Supplementary Data 1.

### Cytokine analysis

Sera of female mice (25-week-old mice (*n* = 7) per group) were isolated from blood samples for cytokine measurement. The levels of cytokines in the sera were measured using the Bio-Plex200 multiplex array system according to the recommended protocol (Bio-Rad, Hercules, CA, USA).

### Sphere formation assay

To facilitate the reformation of spheres, MDA-MB-231 single cells were suspended in serum-free DMEM/F12 medium. The medium was supplemented with antibiotic–antimycotic solution (Invitrogen), B27 (Invitrogen), 10 ng/ml leukemia inhibitory factor (Millipore Ltd., Darmstadt, Germany), 20 ng/ml epidermal growth factor (Invitrogen), and basic fibroblast growth factor (Millipore). The cells were cultured in low attachment plates for 6 days, with medium replenishment every 3 days.

### Proliferation and migration assays

In co-culture with RAW264.7 cells, the proliferation activity of MDA-MB-231 cells was assessed using trans-well chambers with a 4-μm pore size insert (Costar, Cambridge, MA, USA). Briefly, RAW264.7 cells in the upper trans-well chamber and MDA-MB-231 cells in the bottom of 24 well plate were seeded and cultured for 24–48 h. The MDA-MB-231 cell migration assay was assessed in trans-well chambers with an 8-μm pore size insert (Costar). For MDA-MB-231 cell migration in co-culture with RAW264.7 cells, MDA-MB-231 cells and RAW264.7 cells were seeded in the upper trans-well chamber and the bottom of 24 well plate, respectively, and cultured for 24-48 h.

### Immunohistochemistry and analysis of LN metastasis

Mammary glands and tumors excised from 16-week-old female mice (*n* = 5 per group) were fixed with 4% buffered formalin and embedded in paraffin blocks. Next, 4-μm-thick sections were deparaffinized in xylene, rehydrated in a series of graded ethanol and water solutions, and pretreated at 98 °C for 20 min in citrate buffer (pH 6.0) for antigen retrieval. After blocking of endogenous peroxidase activity by peroxidase inhibition buffer and nonspecific binding of immunological reagents by blocking solution (Dako Agilent, CA, USA), each primary antibody was incubated at 4 °C overnight, followed by incubation of HRP-conjugated secondary antibodies, and immunoreaction was visualized using the 3,3′-diaminobenzidine (DAB) chromogen kit (Agilent Technologies, Glostrup, Denmark). Nuclei were counterstained with hematoxylin solution according to the manufacturer's instructions. Hematoxylin and eosin staining were performed.

For analysis of lymph node metastasis in the mammary tumor tissues of PyMT^WT^ and PyMT^SB2−/−^ mice, cancer cells in the lymph nodes were immunostained with an CK8 antibody (Developmental Studies Hybridoma Bank). For the quantification of CK8-immunostained cells, one section per lymph node (*n* = 5) was chosen, and immunostained cells in five fields at 40 × magnification within each section were quantified as the percentage of brown-stained area in each microphotograph using Leica QWin image analysis and image processing software (Leica Imaging Solutions, Cambridge, UK).

### Immunofluorescence staining

For double immunofluorescence staining, additional 4-μm slides of breast cancer tissue from 16-week-old mice were utilized. Primary antibodies, either F4/80 with CD206 or F4/80 with NOS2, were mixed and diluted in antibody diluent (Dako Agilent), and allowed to incubate overnight at 4 °C. The primary antibodies were then visualized with secondary antibodies conjugated to different fluorophores (Alexa Fluor 488 or 594) (Thermo Fisher Scientific) at room temperature for 1 h. Tissue slides were subsequently stained with the 4’,6-diamidino-2-phenylindole dihydrochloride (DAPI) for nuclear counterstaining and covered using Prolong antifade mounting medium (Solarbio, Beijing, China). Immunofluorescence images were captured using an Olympus BX63 fluorescence microscope (Shinjuku-ku, Tokyo, Japan), using identical exposure and gain settings. Mean fluorescence intensity (MFI) of double-stained cells in each tumor section (*n* = 3) was calculated using ImageJ software.

### Analysis of the BreastMark dataset

The prognostic value of the putative genes in patients with breast cancer was determined using the publicly available online tool BreastMark; disease-free survival (DFS) was analyzed by the combination of NOS2, SerpinB2, and CD206 expression, and the median was used to dichotomize the data [19]. N tells us the number of samples used in this comparison. The Hazard ratio (HR) is generated using Cox regression, and a logrank test is used to assign significance to the HR.

### Statistical analysis

All data were expressed as the means ± standard errors (S.E). Statistical significance was determined by unpaired t-test, one-way analysis of variance (ANOVA) followed by Tukey's multiple comparison test or a two-tailed Mann–Whitney U-test. Statistical significance was defined as *P < 0.05, ** P < 0.01, *** P < 0.001. GraphPad Prism v9.2.0 (GraphPad Software Inc., La Jolla, CA, USA) was utilized for all statistical analyses.

## Results

### SerpinB2 expression is not detected in tumors of PyMT^SB2−/−^ mice

**Fig. 1:**
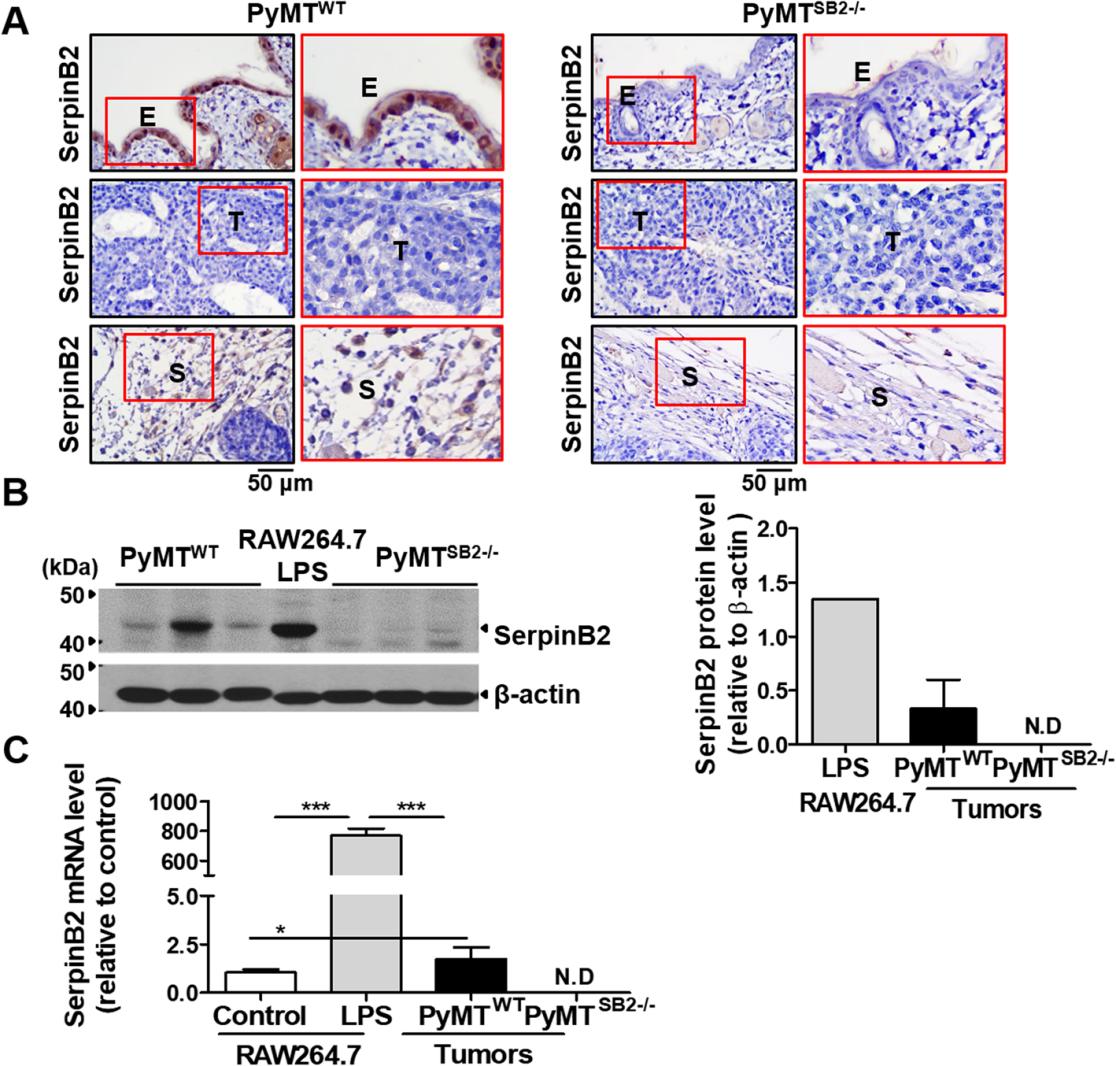
SerpinB2 deficiency expression in mammary tumors. **A** Representative immunostaining images of SerpinB2 in the tumors of PyMT^WT^ and PyMT^SB2−/−^ mice. **B-C **Analysis of SerpinB2 protein and mRNA levels in the tumor lysates of PyMT^WT^ and PyMT^SB2−/−^ mice using western blot and qRT-PCR. Data were presented as the means ± S.E. from the primary tumors of 3 mice per group. *P < 0.05, ***P < 0.001 using unpaired t-test

Our previous studies on mammary tumors in age-matched PyMT^WT^ and PyMT^SB2-/-^ mice demonstrated that SerpinB2 loss delayed tumor onset and significantly reduced tumor incidence rate and volume in the 4th-5th glands in PyMT^SB2−/−^ mice compared to PyMT^WT^ mice [16]. The patterns and expression levels of SerpinB2 in the mammary tumors and stroma of PyMT^WT^ and PyMT^SB2−/−^ mice were analyzed. The abundant expression of SerpinB2 was observed in epidermal keratinocytes of PyMT^WT^ mouse skin, but the tumor cells did not exhibit strong SerpinB2 staining, showing that tumor cells in PyMT^WT^-induced mammary tumors express very low levels of SerpinB2 (Fig. 1A). Intriguingly, stromal cells localized at the peritumoral site of PyMT^WT^ tumors displayed strong SerpinB2-positive staining (Fig. 1A). As expected, significant staining for SerpinB2 was absent in tumors of PyMT^SB2−/−^ mice (Fig. 1A). LPS-treated RAW264.7 cells exhibited high SerpinB2 protein and mRNA levels (Fig. 1B and C). In representative western blot images with quantitative analysis, the SerpinB2 protein levels were low in the tumors of PyMT^WT^ mice (0.41 ± 0.28) but were not detected in the PyMT^SB2−/−^ mice (Fig. 1B). Consistent with the previous western blot, the qRT-PCR analysis revealed no expression of SerpinB2 mRNA in all tumors of PyMT^SB2−/−^ mice (Fig. 1C).

### The SerpinE1 expression levels are higher in PyMT^SB2−/−^ tumors than those in PyMT^WT^ tumors, while there is no significant difference in uPA expression

**Fig. 2:**
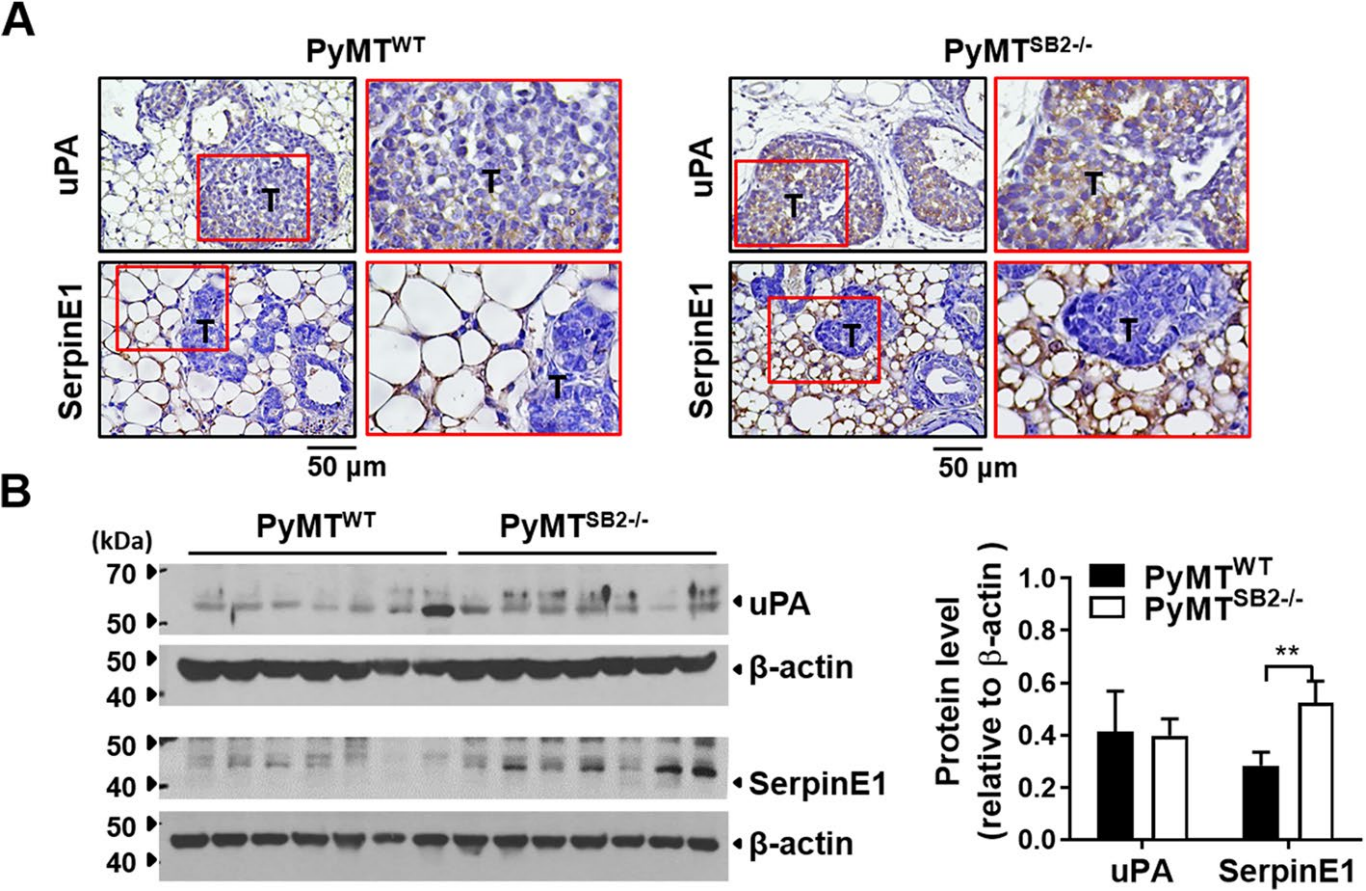
Immunohistochemistry and western blot analysis of uPA and SerpinE1 in tumor tissues. **A** Representative immunostaining images of uPA and SerpinE1 in the tumor tissues of PyMT^WT^ and PyMT^SB2−/−^ mice. **B** Western blot analysis of the uPA and SerpinE1 protein levels in the tumor lysates of PyMT^WT^ and PyMT^SB2−/−^ mice. Data were presented﻿ as the means ± S.E. from tumors of 7 mice per group. **P < 0.01 using unpaired t-test

The expression patterns and levels of uPA and SerpinE1 in the mammary tumors of PyMT^WT^ and PyMT^SB2−/−^ mice were analyzed by immunohistochemistry and western blotting. Strong staining of uPA was observed in the tumor cells of both PyMT^WT^ and PyMT^SB2−/−^ tumor tissues (Fig. 2A). SerpinE1 staining was strong in stromal areas, including adipocytes, fibroblasts, and macrophages, but not in tumor cells in both PyMT^WT^ and PyMT^SB2−/−^ tumor tissues (Fig. 2A). Figure 2B shows a representative western blot for uPA and SerpinE1. SerpinE1 protein level were significantly higher in PyMT^SB2−/−^ tumors (0.54 ± 0.09﻿) than in PyMT^WT^ tumors (0.24 ± 0.03) (*P* = 0.008). In contrast, uPA protein levels exhibited no significant difference between PyMT^WT^ (0.41 ± 0.16) and PyMT^SB2−/−^ (0.40 ± 0.01) tumors (*P* = 0.94) (Fig. 2B).

### Identification of DEGs related to M1/M2 polarization markers in PyMT^WT^ and PyMT^SB2−/−^ tumors

**Fig. 3:**
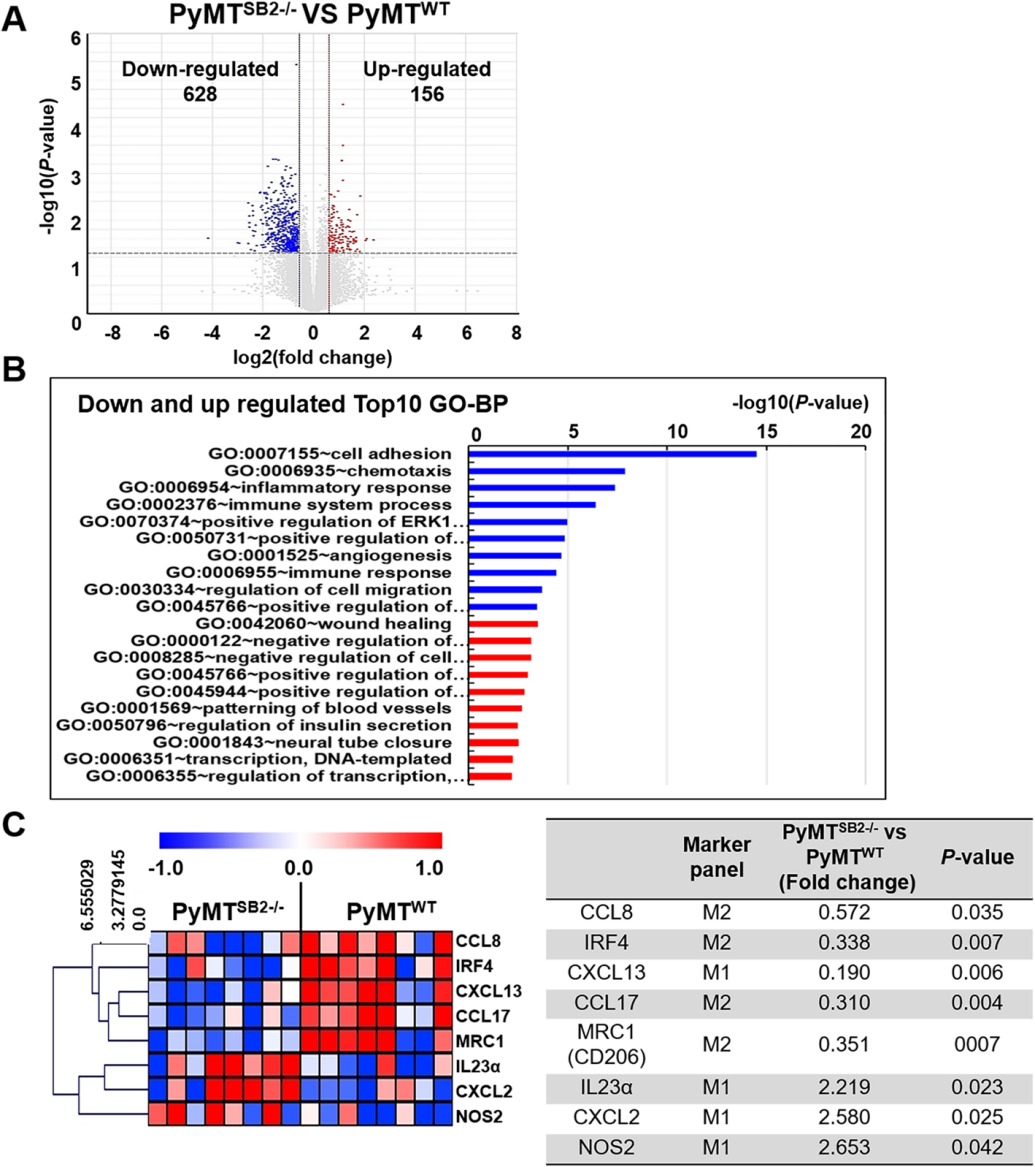
Differentially expressed gene (DEG) clusters and Gene Ontology (GO) categorization of all transcriptomes assessed in the RNA-Seq analysis of primary tumors of PyMT^WT^ and PyMT^SB2−/−^ mice. **A** Volcano plot of the relationship between fold-change and significance between the two groups. **B** Top 10 enriched GO terms in the significantly up and downregulated DEGs in PyMT^SB2−/−^ tumors relative to PyMT^WT^ tumors. **C** Heatmap reflecting the expression profiles of DEGs associated with macrophage polarization

After RNA-Seq transcriptional profiling analysis of PyMT^WT^ and PyMT^SB2−/−^ tumors, 23,282 expressed genes were identified. Compared with the PyMT^WT^ tumors, 784 DEGs were identified in PyMT^SB2−/−^ tumors. Of the 784 genes, 156 were upregulated and 628 were downregulated (Fig. 3A). In the top 10 enriched GO terms of biological process in up and downregulated DEGs of PyMT^SB2−/−^ tumors compared to PyMT^WT^ tumors, we found the following GO terms: wound healing, negative regulation of cell proliferation, cell adhesion, inflammatory response and immune system process (Fig. 3B and Table S2). DEGs (CCL8, IRF4, CXCL13, CCL17, CD206, IL23α, CXCL2, NOS2) related to twofold increased M1 macrophage markers and twofold decreased M2 macrophage markers were identified in the primary tumors of PyMT^SB2−/−^ mice relative to those of PyMT^WT^ mice. However, the expression of CXCL13, a marker of M1 macrophages, was decreased fivefold in PyMT^SB2−/−^ mice relative to those of PyMT^WT^ mice (Fig. 3C).

### M2 marker CD206 decreases whereas the M1 marker NOS2 increases in the TAMs localized in PyMT^SB2−/−^ tumors

**Fig. 4:**
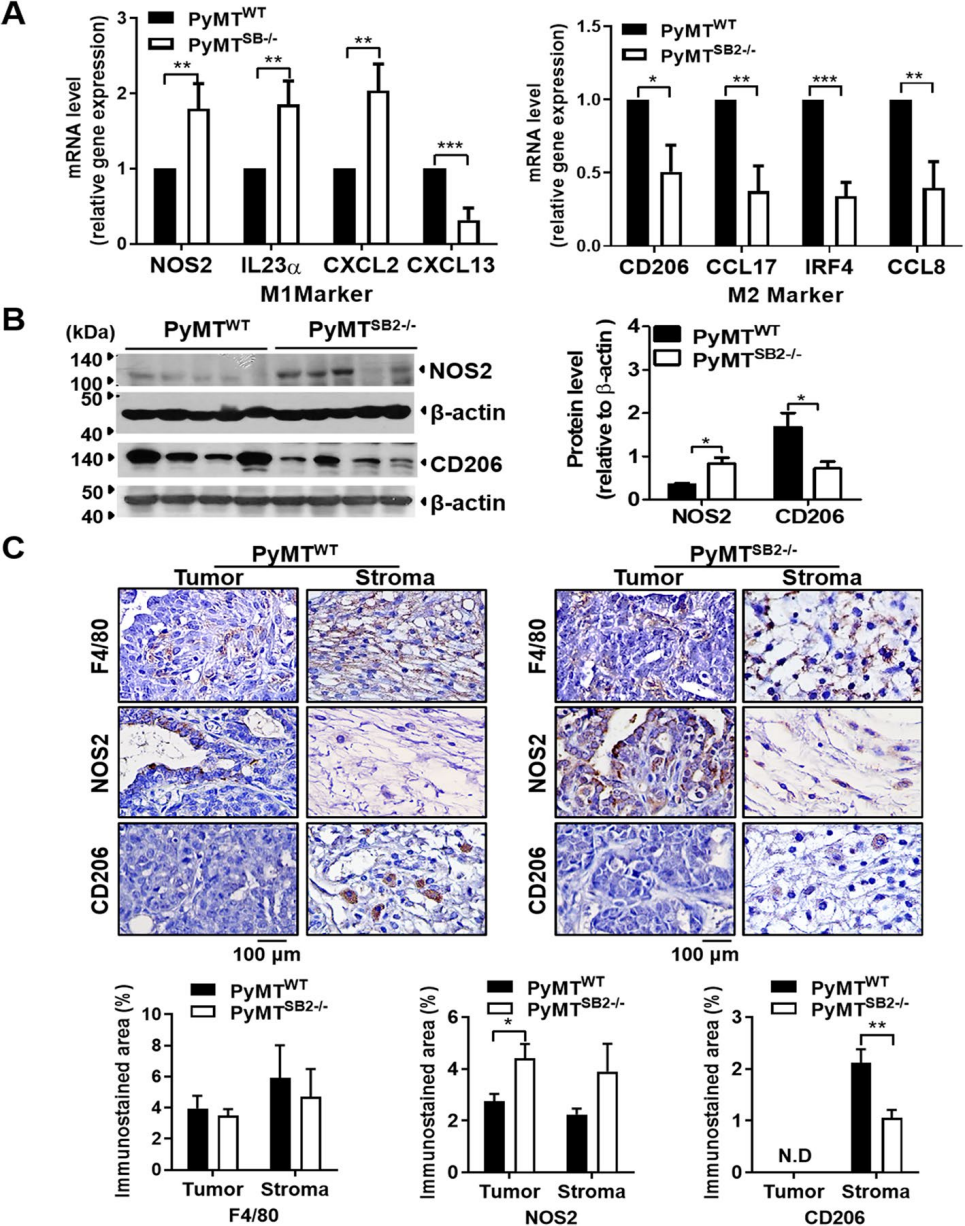
SerpinB2 deficiency results in increased NOS2^+^M1 and decreased CD206^+^M2 macrophage polarization. **A **Analysis of relative mRNA expressions of M1 polarization markers (NOS2, IL23α, CXCL2, CXCL13) and M2 polarization markers (CD206, CCL17, IRF4, CCL8) in the primary tumors of PyMT^WT^ and PyMT^SB2−/−^ mice using qRT-PCR. Data were presented﻿ as means ± S.E. of primary tumors of 8 mice per group. **B** Western blot analysis of the NOS2 and CD206 protein levels in the tumor lysates of PyMT^WT^ and PyMT^SB2−/−^ mice. Data were presented as the means ± S.E. from tumors of 4–5 mice per group. **C** Immunostaining analysiss of F4/80, NOS2, and CD206 in the tumor tissues of PyMT^WT^ and PyMT.^SB2−/−^ mice. Data were presented as the means ± S.E. from tumor sections of 5 mice per group. *P < 0.05, **P < 0.01, ***P < 0.001 using unpaired t-test

The expression levels of M1 and M2 related DEGs in PyMT^WT^ and PyMT^SB2−/−^ tumors were validated by qRT-PCR. As shown in Fig. 4A, M1 macrophage markers NOS2 (1.80 ± 0.14, *P* = 0.01), IL23α (1.85 ± 0.14, *P* = 0.004), and CXCL2 (2.04 ± 0.15, *P* = 0.003) were significantly increased, while M2 macrophage markers CD206 (0.51 ± 0.11, *P* = 0.03), CCL17 (0.37 ± 0.10, *P* < 0.005), IRF4 (0.34 ± 0.05, *P* < 0.001), and CCL8 (0.40 ± 0.10, *P* = 0.005) were significantly decreased in PyMT^SB2−/−^ tumors compared to PyMT^WT^ tumors. Conversely, the mRNA of CXCL13 (0.32 ± 0.09, *P* < 0.001) exhibited a significant decrease in PyMT^SB2−/−^ tumors compared to PyMT^WT^ tumors, as demonstrated in the heatmap. Based on the comparison of M1- and M2-associated genes by RNA-Seq and qRT-PCR, we focused on validating the expression of NOS2 and CD206 in the tumor tissues. Western blot analysis of NOS2 and CD206 obtained from the tumors of 25-week-old PyMT^WT^ and PyMT^SB2−/−^ mice revealed that the CD206 protein level was lower in PyMT^SB2−/−^ (0.72 ± 0.16) than that in PyMT^WT^ tumors (1.67 ± 0.33) (*P* = 0.043), whereas the NOS2 protein level was comparably higher in PyMT^SB2−/−^ (0.83 ± 0.14) than found in PyMT^WT^ tumors (0.34 ± 0.03) (*P* = 0.01) (Fig. 4B).

**Fig. 5:**
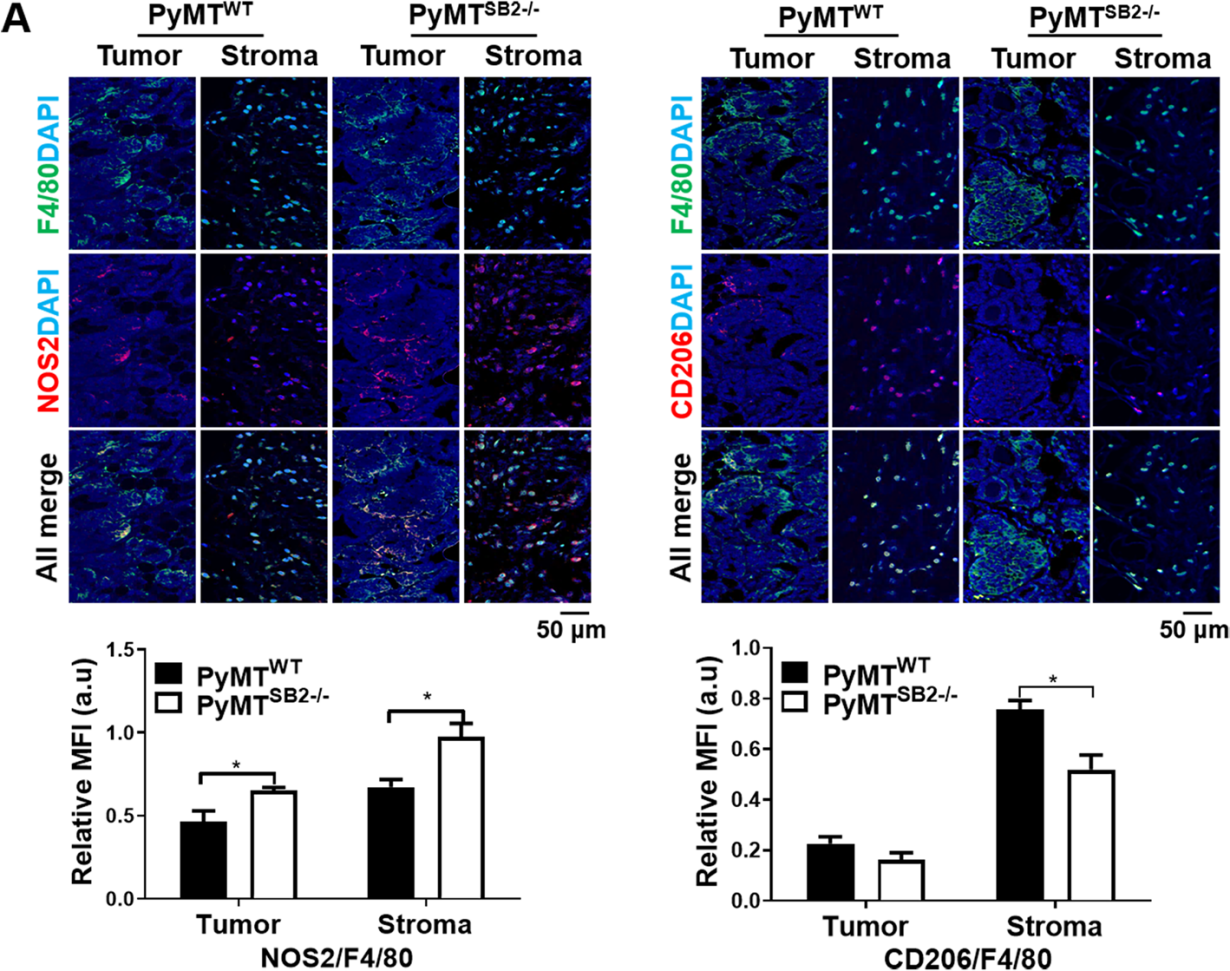
NOS2 + F4/80 + M1 macrophages were more abundant than CD206 + F4/80 M2 macrophages in PyMT^SB2−/−^ tumors compared to PyMT^WT^ tumors. **A** Representative double immunofluorescence staining images and quantification of mean fluorescence intensity (MFI)﻿ of F4/80, NOS2 or CD206 in the peritumoral stroma and intratumoral area of PyMT^WT^ and PyMT^SB2−/−^ tumor tissues. Staining for macrophage markers F4/80 (green); M1 marker, NOS2 (red); M2 marker, CD206 (red); nuclei, DAPI (blue). Double positive NOS2 + F4/80 + (yellow) and CD206 + F4/80 + cells (yellow) in the tumor sections of PyMT^WT^ and PyMT^SB2−/−^ mice. Data were presented as the means ± S.E. from 3 sections per group. *P < 0.05 using unpaired t-test

Immunohistochemistry for F4/80, NOS2, and CD206 was performed to investigate the TAM population and polarized phenotype localized in the intratumoral areas and peritumoral stroma of each tumor in 16-week-old PyMT^WT^ and PyMT^SB2−/−^ mice. A large population of macrophages detected by the F4/80 monoclonal antibody accumulated in the intratumoral area (3.49 ± 0.41 vs 3.95 ± 0.81, *P* = 0.60) as well as in the peritumoral stroma (4.70 ± 1.79 vs 5.92 ± 2.09, *P* = 0.68) of both mice (Fig. 4C, top). The NOS2-positive cell population was more abundant in the intratumoral area (4.40 ± 0.56 vs 3.01 ± 0.36, *P* = 0.036) and peritumoral stroma (3.89 ± 1.07 vs 2.47 ± 0.30, *P* = 0.156) of PyMT^SB2−/−^ mice than in those of PyMT^WT^ mice (Fig. 4C, middle). The CD206-positive cell population was substantially decreased in the peritumoral stroma (1.05 ± 0.15 vs 2.12 ± 0.26, *P* = 0.003) of PyMT^SB2−/−^ mice compared with that in PyMT^WT^ mice and was not observed in the intratumoral area of both PyMT^WT^ and PyMT^SB2−/−^ mice (Fig. 4C, bottom). In addition, double immunofluorescence staining with F4/80 and NOS2 or CD206 was performed to identify TAMs that express NOS2 or CD206. The double-positive F4/80 + NOS2 + TAMs were more abundant in both the peritumoral stroma (0.67 ± 0.04 vs 0.98 ± 0.08, *P* = 0.029) and intratumoral regions (0.46 ± 0.06 vs 0.65 ± 0.02, *P* = 0.049) of PyMT^SB2−/−^ tumor compared to those of PyMT^WT^ tumors. Conversely, the double-positive F4/80 + CD206 + TAMs notably decreased in both the peritumoral stroma (0.76 ± 0.04 vs 0.52 ± 0.06, *P* = 0.026), and intratumoral regions (0.03 ± 0.03 vs 0.16 ± 0.03, *P* = 0.18) (Fig. 5A), which was consistent with the immunohistochemistry results.

### CK8-positive tumor cells and CD206-positive TAMs decrease in metastatic LNs of PyMT^SB2−/−^ mice

**Fig. 6:**
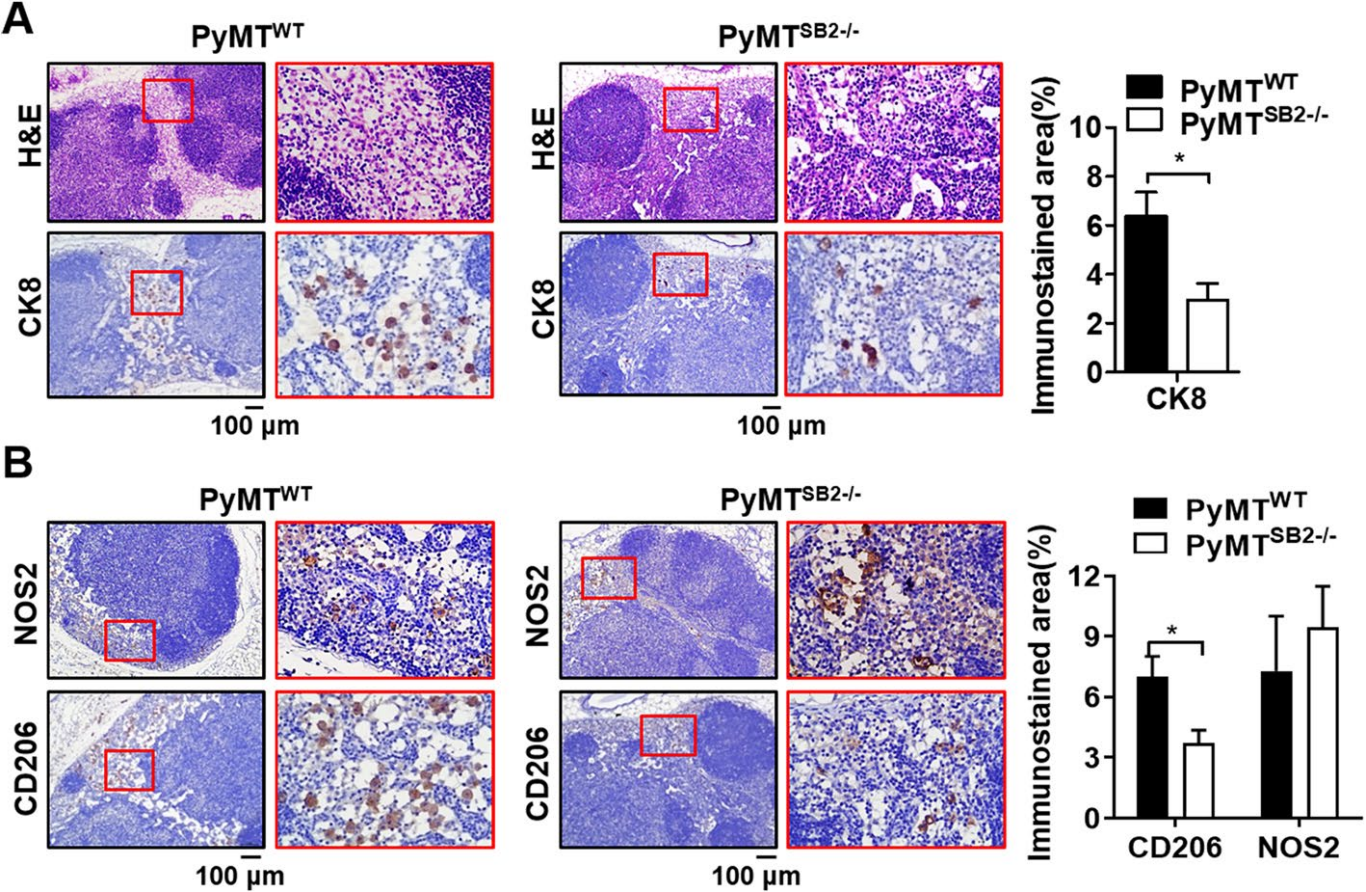
SerpinB2 deficiency reduces tumor cell dissemination to the lymph nodes. **A** H&E staining and CK8 immunostaining for detecting metastatic tumor cells in the lymph nodes of the 4th mammary gland in PyMT^WT^ and PyMT^SB2−/−^ mice. **B** NOS2 and CD206 immunostaining in the metastatic lymph nodes of PyMT^WT^ and PyMT^SB2−/−^ mice. Immunohistological analysis obtained from the lymph nodes of 5 mice per group were expressed as the mean ± S.E. *P < 0.05 using two-tailed Mann–Whitney U-test ﻿

To test the intrinsic role of SerpinB2 in LN metastasis, local LN metastasis in 16-week-old PyMT^WT^ and PyMT^SB2−/−^ mice with comparable mammary tumors was analyzed by CK8 immunostaining. The incidence of LN metastasis was 100% in PyMT^WT^ and PyMT^SB2−/−^ mice bearing tumors. Figure 6A (top) shows the H&E staining of LNs in the 4th mammary tumors of PyMT^WT^ and PyMT^SB2−/−^ mice. Representative immunohistochemistry of the epithelial marker CK8 revealed a reduction in number of CK8-positive tumor cells within the trabecular zone of LN sections of PyMT^SB2−/−^ mice (2.95 ± 0.67%) compared with that of PyMT^WT^ mice (6.37 ± 0.98%) (Fig. 6A bottom and Table 1, *P* = 0.02).

**Table 1:** Analysis of regional LN metastasis and macrophage polarization by immunohistochemistry of CK8, NOS2, and CD206 in the LNs of 16-week-old PyMT^WT^ and PyMT^SB2−/−^ mice

	**PyMT**^**WT**^ **(*n* = 5)**	**PyMT**^**SB2−/−**^ **(*n* = 5)**	***P*-value**
CK8^+^area (%)	6.37 ± 0.98	2.95 ± 0.67	0.0201
NOS2^+^area (%)	7.29 ± 2.72	9.45 ± 2.03	0.5408
CD206^+^area (%)	7.01 ± 1.00	3.71 ± 0.66	0.0252

The data are represented as the means ± S.E. from the lymph nodes of tumors of 5 mice per group. Statistical analysis by two-tailed Mann–Whitney U-test

Next, the TAM phenotypic polarization status in LN metastasis was investigated by immunohistochemistry of NOS2 and CD206. As shown in Fig. 6B, TAMs were detected in the trabecular zone of LNs, and the NOS2-positive area was increased, but there was no statistically significant difference in the LNs of PyMT^SB2−/−^ mice (9.45 ± 2.03%) compared with that in PyMT^WT^ mice (7.29 ± 2.72%). A significantly decreased CD206-positive area was found in the LNs of PyMT^SB2−/−^ mice (3.71 ± 0.66%) relative to that of PyMT^WT^ mice (7.01 ± 1.00%) (Fig. 6B and Table 1,* P* = 0.025).

The levels of IL-2, IL-4, IL-5, IL-6, IL-10, IL-12, IL-13, GM-CSF, IFN-γ, and TNF-α associated with cancer immunity in the serum samples of peripheral blood of PyMT^WT^ mice were comparably higher relative to those in PyMT^SB2−/−^ mice (Table S3).

### SerpinB2 knockdown decreases the sphere formation and migration of MDA-MB-231 cells and results in low CD206 and high NOS2 expression in RAW264.7 cells

**Fig. 7:**
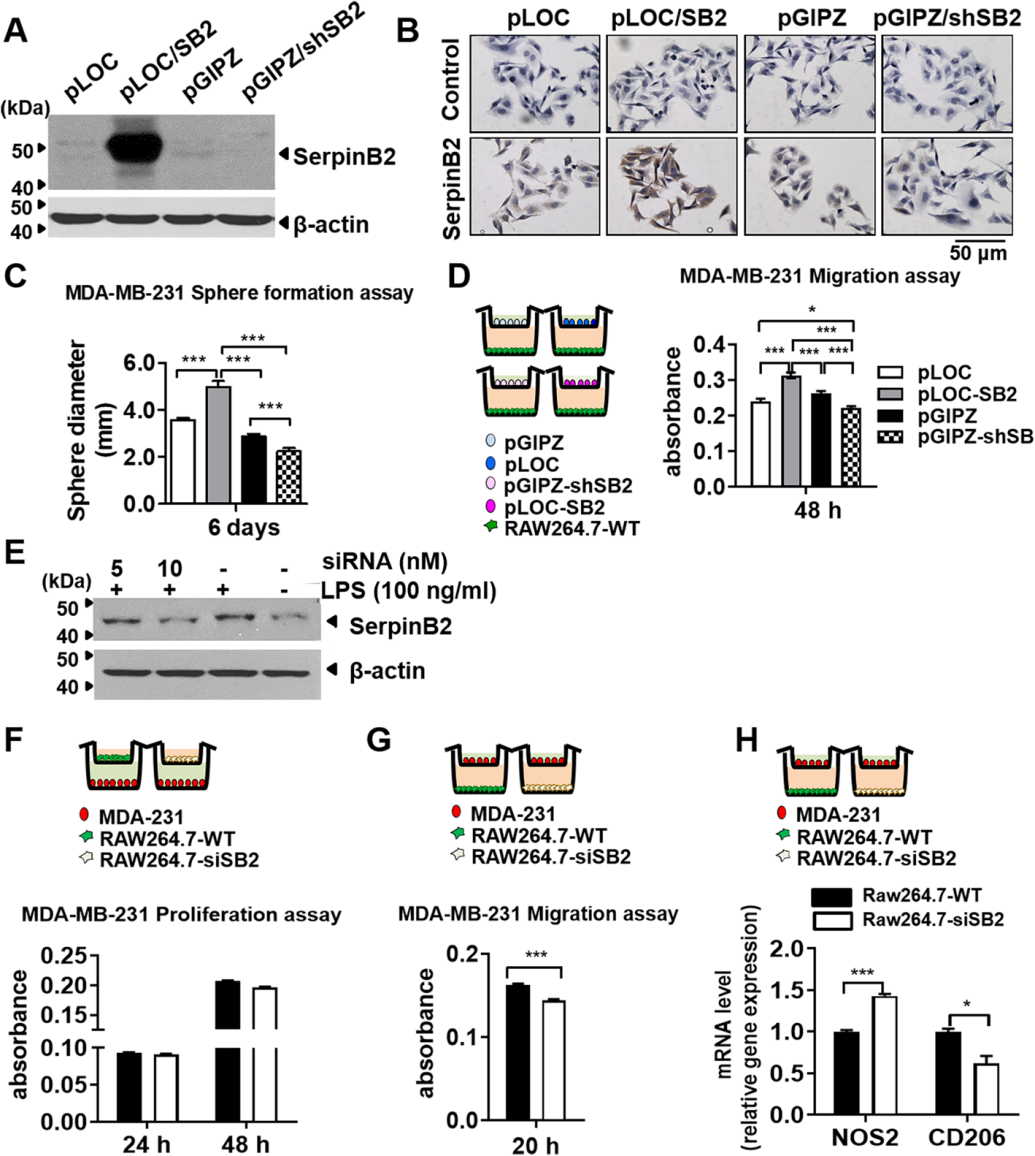
SerpinB2 knockdown reduces the sphere formation and migration of breast cancer cells and results in NOS2 upregulation and CD206 downregulation in macrophages. **A-B** Western blot and immunocytochemistry of SerpinB2 in SerpinB2-knockdown or -overexpressed MDA-MB-231 cells using pGIPZ/shSB2 or pLOC/SB2 lentivirus. **C** Sphere forming assay of pGIPZ/shSB2 or pLOC/SB2 MDA-MB-231 cells. **D** Migration assay of SerpinB2-knockdown or -overexpressed MDA-MB-231 cells co-cultured with wild type RAW264.7 cells (RAW264.7-WT)**. E** Western blot of SerpinB2 in LPS-treated or siRNA-mediated SeripinB2 knockdown RAW264.7 cells (RAW264.7-siSB2). **F-G** Proliferation and migration assays of MDA-MB-231 cells co-cultured with RAW264.7-siSB or RAW264.7-WT cells. **H** qRT-PCR analysis of NOS2 and CD206 in RAW264.7-siSB or RAW264.7-WT cells co-cultured with MDA-MB-231 cells. All experiments were performed in triplicate for each condition and repeated at least three times. Data were presented as the means ± S.E. *P < 0.05, ***P < 0.001 using unpaired t-test or one-way analysis of variance (ANOVA) followed by Tukey's multiple comparisons test﻿

We previously reported that SerpinB2 upregulated by microRNA 200c/141 in MDA-MB-231 cells was involved in promoting lung metastasis [4]. To examine whether tumor cell-produced SerpinB2 is responsible for the proliferation and migration of breast cancer cells, the SerpinB2 gene (pLOC-SB2) or shRNA (pGIPZ-shSB2) was transduced into MDA-MB-231 cells using lentivirus (Fig. 7A). In the immunocytochemistry analysis of SerpinB2, SerpinB2 was substantially detected in the cytoplasm of the SerpinB2 overexpression cell line MDA-MB-231-pLOC/SB2, while detection was nearly negligible in the SerpinB2-knockdown cell line MDA-MB-231-pGIPZ/shSB2 (Fig. 7B). The sphere diameters significantly decreased in SerpinB2 knockdown MDA-MB-231 cells (pGIPZ-shSB2, 2.24 ± 0.13 mm) compared with the cells stably overexpressing SerpinB2 (pLOC-SB2, 4.98 ± 0.24 mm) and control vectors (pGIPZ, 2.87 ± 0.11 mm and pLOC, 3.55 ± 0.096 mm) (Fig. 7C, *P* < 0.0001). In co-culture with RAW264.7 cells, the migration ability of pGIPZ-shSB2 (0.22 ± 0.005) significantly decreased compared with that of pLOC-SB2 (0.31 ± 0.008) and control vectors (pGIPZ, 0.26 ± 0.007 and pLOC, 0.24 ± 0.007) (*P* < 0.0001 Fig. 7D).

Next, to explore the function of SerpinB2 in M1/M2 macrophage polarization and tumor cell proliferation and migration, SerpinB2 siRNA-transfected RAW264.7 (RAW264.7-siSB2) or wild-type RAW264.7 cells (RAW264.7 WT) were co-cultured with MDA-MB-231 cells. LPS induced the upregulation of SerpinB2, but SerpinB2 siRNA (10 nM) suppressed the LPS-induced SerpinB2 upregulation in RAW264.7 cells (Fig. 7E), indicating effective knockdown of SerpinB2. The non-targeting siRNA (scramble) had no discernible impact on the expression of SerpinB2 in macrophages (Supplementary Data 2A). Co-culturing with SerpinB2 knockdown RAW264.7 cells (RAW264.7-siSB2) significantly reduced MDA-MB-231 cell migration (0.14 ± 0.001 vs 0.16 ± 0.002, *P* < 0.0001) but not proliferation (Fig. 7F and 7G). In co-culture of MDA-MB-231 cells, RAW264.7-siSB2 exhibited significantly higher NOS2 mRNA levels (9.20 ± 0.03 vs 8.68 ± 0.02, *P* = 0.0002) and lower CD206 mRNA levels (14.07 ± 0.05 vs 14.8 ± 0.23,* P* = 0.037) than RAW264.7-WT (Fig. 7H). Notably, there were no significant differences in the mRNA levels of NOS2 and CD206 between RAW264.7-WT and RAW264.7-siScramble (Supplementary Data 2B).

### Combination of low SerpinB2, high NOS2, and low CD206 expression is a prognostic indicator of favorable survival

**Fig. 8:**
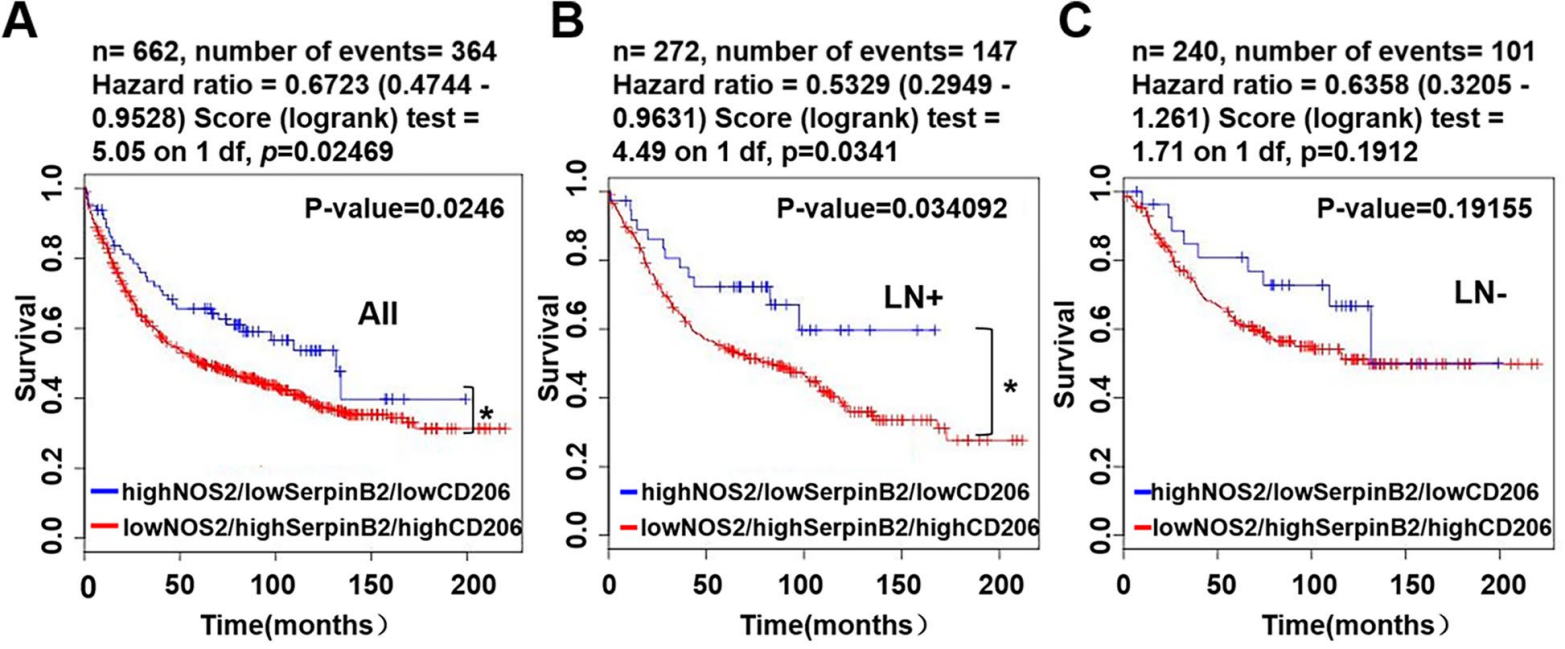
Kaplan–Meier plots of breast cancer patient survival based on the combination of SerpinB2, NOS2, and CD206 expression in the BreastMark dataset. **A** Disease-free survival (DFS) analysis of all breast cancer patients using the combination of three genes. **B-C** DFS analysis of breast cancer patients with or without lymph node (LN) metastasis using the combination of three genes

The BreastMark website was used to explore the association between SerpinB2, NOS2, and CD206 expression, and DFS in patients with breast cancer. The samples were separated into the combination of low SerpinB2, high NOS2, and low CD206 groups and the combination of high SerpinB2, low NOS2, and high CD206 groups. Kaplan–Meier plots revealed that patients with the combination of low SerpinB2, high NOS2, and low CD206 expression exhibited a significantly favorable DFS (*n *= 662, HR = 0.6723, *P* = 0.02469, Fig. 8A). The combination of low SerpinB2, high NOS2, and low CD206 expression was associated with good DFS in patients with breast cancer with LN metastasis (*n *= 272, HR = 0.5329, *P* = 0.0341, Fig. 8B). The DFS in patients with breast cancer without LN metastasis (*n* = 240, HR = 0.6358, *P* = 0.1912) was not significantly different, but there was still a notable difference in DFS within 132 months (11 years) in patients with breast cancer without LN metastasis (Fig. 8C). The analyses of the combinations of SerpinB2/NOS2, SerpinB2/CD206, and NOS2/CD206 groups, as well as the individual expressions of SerpinB2, NOS2, or CD206 alone did not demonstrate a significant difference in the DFS of patients with breast cancer (Supplementary Data 3).

## Discussion

The role of SerpinB2 as a prognostic marker in breast cancer progression or suppression remains controversial. This study represents the first trial to analyze the consequences of SerpinB2 deficiency on tumorigenesis, tumor growth, and metastasis throughout the course of mammary cancer progression. We show that in PyMT^SB2−/−^ mice, SerpinB2 deficiency delays mammary tumor initiation, growth, and LN metastasis, which is accompanied by a decrease in CD206^+^M2 TAMs and an increase in NOS2^+^M1 TAMs.

Although SerpinB2 expression was observed in diverse cell types, the significant role of SerpinB2 expressed in TAMs as well as tumor cells during mammary tumor development and progression remains poorly understood. Several mechanisms have been proposed where SerpinB2 expression by tumor cells might influence tumorigenesis and include the inhibition of apoptosis or uPA signaling [3, 20, 21]. The reports revealed that SerpinB2 expression in tumor cells may function as a survival factor by repressing proapoptotic signal transduction. We previously found that SerpinB2-upregulated MDA-MB-231 cells that were stably overexpressing miR200c promoted lung metastasis and boosted macrophage infiltration in tumor tissues, but the knockdown of SerpinB2 decreased lung metastasis and macrophage infiltration in xenograft mouse models [4]. Current study revealed that the knockdown of SerpinB2 in tumor cells led to a notable decrease in the sphere formation and migration of MDA-MB-231 cells and delayed mammary cancer progression and LN metastasis in PyMT^SB2−/−^ mice, suggesting that mammary tumor cell-produced SerpinB2 may contribute to the aggressiveness on of mammary tumor cells.

SerpinE1 and SerpinB2, both serine protease inhibitors, regulate the activity of plasminogen activators, primarily uPA [22]. Their role in breast cancer appears to be paradoxical, with both pro-tumorigenic and potential tumor-suppressive effects: they can promote tumor progression by facilitating diverse signaling pathways involved in inflammation, cell proliferation, angiogenesis, and invasion, in certain contexts, whereas they have anti-tumor effects by inhibiting plasminogen activators and reducing metastatic potential [2, 22, 23]. Compensatory mechanisms involving SerpinE1 and SerpinB2 have implications in various diseases, including cancer [24]. In a previous study, we found that miR200c/141-transduced MDA-MB-231 cells had higher SerpinB2 but lower SerpinE1 levels [4]. In present findings, SerpinB2 deficiency in PyMT^SB2−/−^ tumors led to a significant increase in SerpinE1 expression, but no significant change in uPA expression. This suggests as SerpinB2 expression decreases, SerpinE1 expression may increase to compensate for their role in regulating uPA activity. SerpinE1, which is upregulated in PyMT^SB2−/−^ tumors, may inhibit uPA activity, potentially suppressing uPA-mediated breast cancer development and progression. Therefore, understanding the intricate interplay among SerpinB2, SerpinE1 and uPA is crucial for unraveling their roles in breast cancer biology and developing targeted therapeutic strategies.

SerpinB2 is one of the most strongly induced proteins in macrophages during infection and inflammation and is emerging as a novel regulator in the macrophage immune response [9, 14, 23]. SerpinB2 protein levels are higher in M2 than in M1 macrophages [12]. In our previous study of transcriptome analysis of mammary tumors of PyMT^SB2−/−^ mice, the expression levels of cancer immune modulated genes (ANXA3, CCL17, CXCL13, CXCR3, IFN-γ, NR4A1, and SEMA3a) were significantly changed [16]. Among these genes, CCL17, CXCL13, and SEMA3a, which tend to polarize macrophages into M2 type or are secreted by M2 macrophages, were significantly reduced in PyMT^SB2−/−^ tumors [24–26].

Based on co-culture studies with MDA-MB-231 and SerpinB2-knockdown RAW264.7 (RAW264.7-siSB2), we speculate that macrophage-produced SerpinB2 regulates the migration ability of breast cancer cells without substantially impacting proliferative activity, leading to increased metastasis. The following observations support our finding: an increase in NOS2-positive M1 macrophages and a decrease in CD206-positive M2 macrophages in the tumor tissues of PyMT^SB2−/−^ mice and the SerpinB2 knockdown RAW264.7 cells. We previously found that SerpinB2-upregulated MDA-MB-231 cells that were stably overexpressing miR200c promoted lung metastasis and boosted macrophage infiltration in tumor tissues, but the knockdown of SerpinB2 decreased lung metastasis and macrophage infiltration in xenograft mouse models [4], implying that SerpinB2 secreted by MDA-MB-231 cells may contribute to macrophage migration and polarization. Recently, Meng et. al reported that SerpinB2 in MDA-MB-231 cells with miR-200c overexpression increased the secretion of IL-10, resulting in the induction of M2 polarization of RAW264.7 cells [27], which supports our findings. Although we investigated the NOS2 and CD206 known as M1 and M2 polarization markers typically expressed in both human and mouse macrophages in cross-talk experiments between MDA-MB-231 and RAW264.7, there are potential caveats such as a species mismatch between the cell lines and discrepancies in M1 and M2 polarization markers between human and mouse macrophages [28]. Taken together, our results suggest that SerpinB2 deficiency leads to macrophages with more M1 and fewer M2 characteristics, resulting in an inhibitory effect on the growth and metastatic potential of mammary cancer. SerpinB2 deficiency is associated with a dysregulation of the Th1- and Th2-promoting cytokine release [9, 14, 23], which has traditionally been viewed as inhibiting and favoring tumor growth. We found that the circulating levels of Th1 and Th2 cytokines in the peripheral blood of tumor-bearing PyMT^SB2−/−^ mice were lower than those in PyMT^WT^ mice. These findings imply that SerpinB2 deficiency may cause Th1/Th2 immune perturbations in PyMT^SB2−/−^ mice. Further research is needed to fully understand the molecular mechanisms and clinical implications of the interplay between SerpinB2 and Th1/Th2 immune responses in breast cancer.

SerpinB2 function remains paradoxical in breast cancer; SerpinB2 is associated with reduced metastasis and prolonged survival in patients with breast cancer [29, 30] and shows increased significance for a favorable prognosis [31–33]. In contrast, SerpinB2 promotes tumorigenesis and provides metastatic potential by inhibiting apoptosis and fostering vascular co-option advantage [3, 4, 21]. In our previous study, immunohistochemistry revealed that SerpinB2 protein is highly expressed in tumor cells in aggressive breast tumor tissue with the triple-negative subtype, and is associated with a short overall survival in breast cancer patients [4]. The BreastMark dataset based on mRNA analysis showed no significant difference in DFS between breast cancer groups with low and high SerpinB2 expression. The inconsistent results for survival between our previous report and the BreastMark data might be due to differences in experimental analysis; thus, our previous study demonstrated the SerpinB2 expression level by immunohistological analysis in tumor cells. We found that the combination of low SerpinB2, high NOS2, and low CD206 mRNA expression was associated with favorable DFS in patients with breast cancer, suggesting that rather than focusing on a single gene biomarker, a combined multigene marker may have more powerful prognostic or predictive value.

## Conclusion

Our findings reveal that in vivo SerpinB2 deficiency delays breast cancer development and metastasis and decreases the differentiation of TAMs toward a protumorigenic M2 phenotype. The combination of SerpinB2, NOS2, and CD206 expression in tumor cells as well as TAMs and its clinical significance should be further evaluated in a cohort of patients with breast cancer.

### Supplementary Information

Supplementary Material 1.

Supplementary Material 2.

Supplementary Material 3.

Supplementary Material 4.

Supplementary Material 5.

Supplementary Material 6.

## Data Availability

The raw transcriptome sequencing data (RNA-Seq) has been publicly released and is available for access in the BioProject database under the accession number PRJDB13249. Other datasets used and/or analyzed during the current study are available from the corresponding author on reasonable request.

## Abbreviations

MMTV: Mouse mammary tumor virus
PyMT: Polyomavirus middle T antigen
PyMT^SB2−/−^: SerpinB2-deficient PyMT
WT: Wild-type
PAI-2: Plasminogen activator inhibitor-2
tPA: Tissue plasminogen activator
uPA: Urokinase plasminogen activator
TAM: Tumor-associated macrophage
NOS2: Marker nitric oxide synthase 2
LN: Lymph node
CK8: Cytokeratin 8
H&E: Hematoxylin and eosin
DFS: Disease-free survival
RNA-Seq: RNA-Sequencing
DEG: Differentially expressed gene
