# Supplementary material for: SerpinB2 deficiency is associated with delayed mammary tumor development and decreased pro-tumorigenic macrophage polarization
Source: BMC Cancer. 2024 Jul 3;24:792. doi: 10.1186/s12885-024-12473-6 (PMC11221169; doi:10.1186/s12885-024-12473-6)

**Original western blot images of Fig. 1B**

**
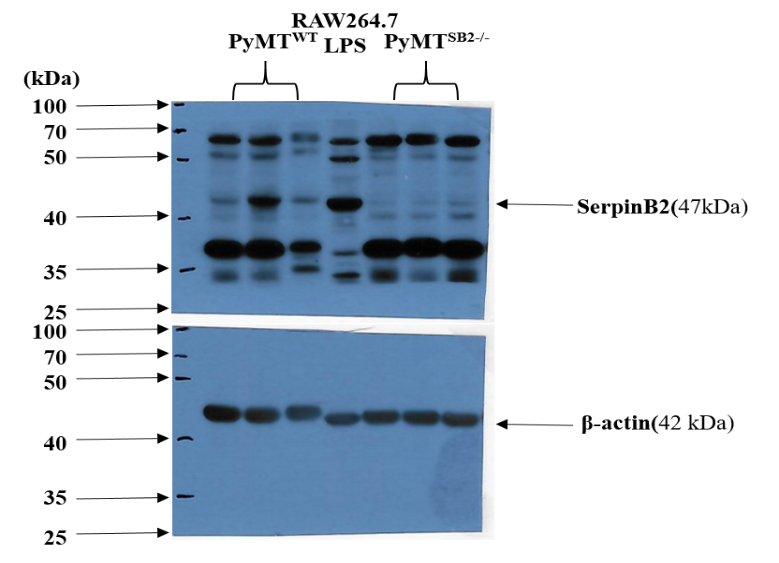
**

**Original western blot images of Fig. 2B**


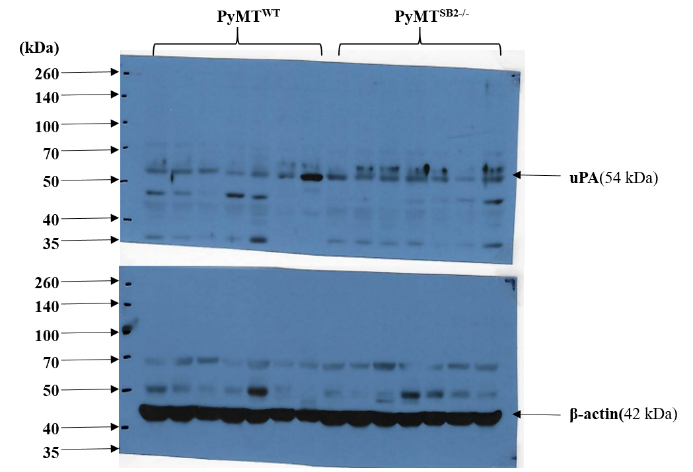


**Original western blot images of Fig. 2B**


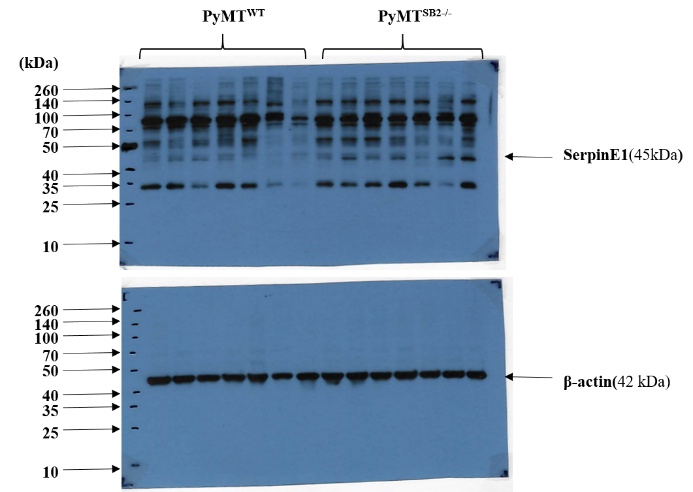


**Original western blot images of Fig. 4B**


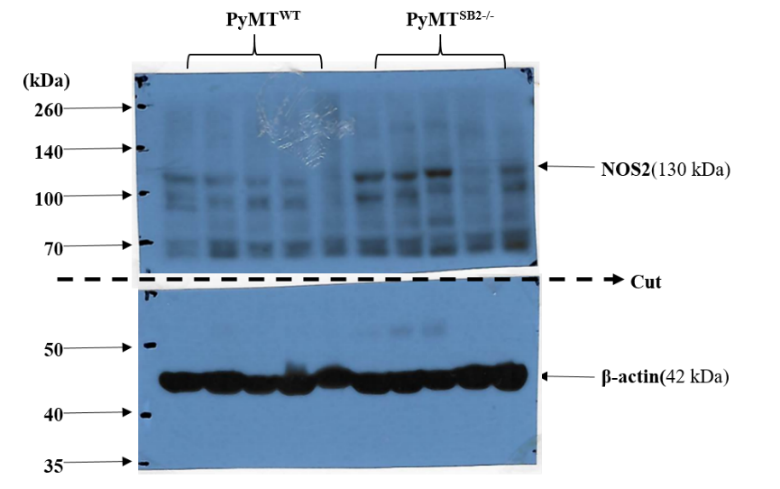


**Original western blot images of Fig. 4B.**


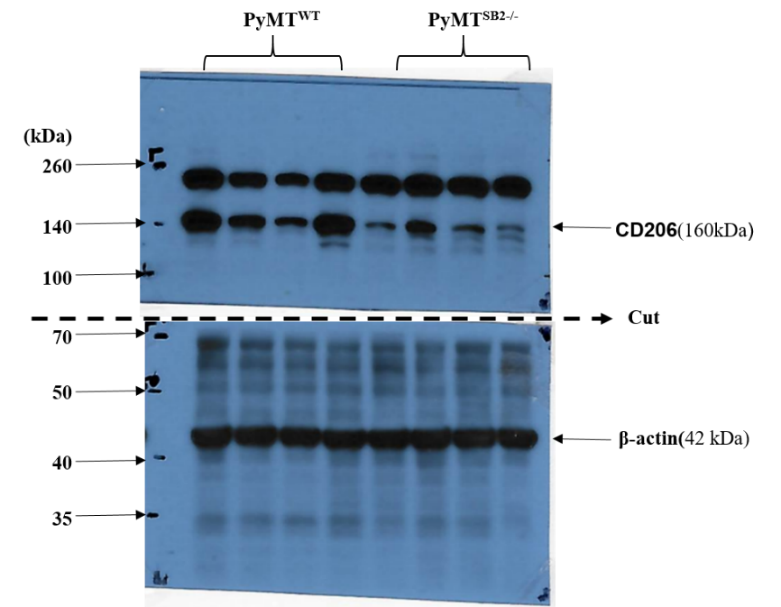


**Original western blot images of Fig. 7A.**


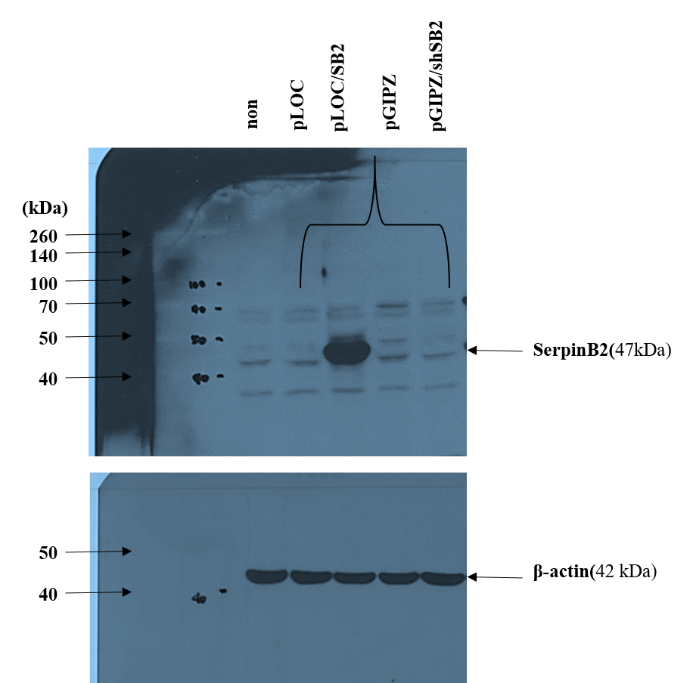


**Original western blot images of Fig. 7E.**


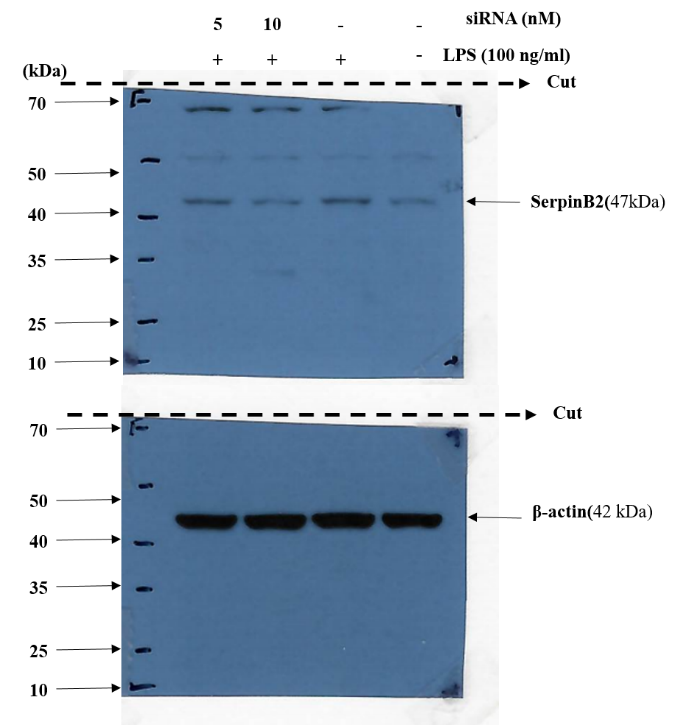

Supplement: Supplementary file 1 — Supplementary Material 1. [file 12885_2024_12473_MOESM1_ESM.docx]
