# Supplementary material for: SerpinB2 deficiency is associated with delayed mammary tumor development and decreased pro-tumorigenic macrophage polarization
Source: BMC Cancer. 2024 Jul 3;24:792. doi: 10.1186/s12885-024-12473-6 (PMC11221169; doi:10.1186/s12885-024-12473-6)
Supplement: Supplementary file 2 — Supplementary Material 2. [file 12885_2024_12473_MOESM2_ESM.docx]

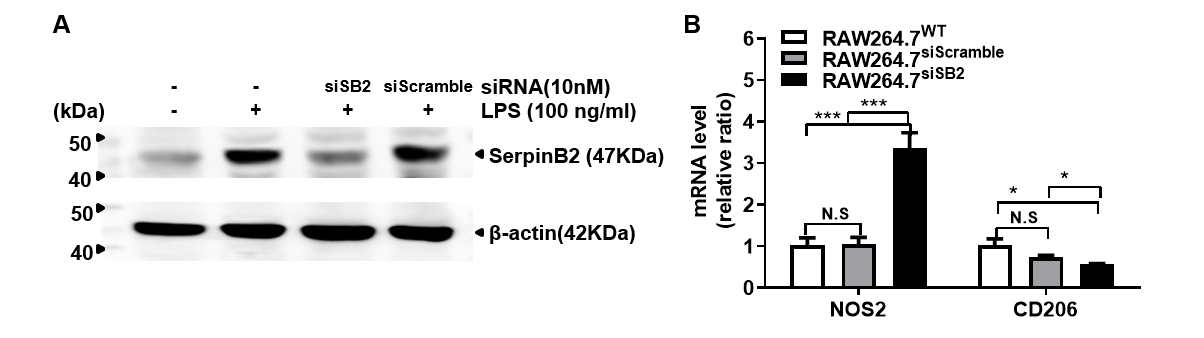


**Supplementary Data 2:** **The non-targeting siRNA had no discernible impact on SerpinB2 expression or the inflammatory response in macrophages. A** Western blot of SerpinB2 in LPS-treated, non-targeting siRNA treated RAW264.7 cells (RAW264.7-siScramble) or siRNA-mediated SeripinB2 knockdown RAW264.7 cells (RAW264.7-siSB2). **B** qRT-PCR analysis of NOS2 and CD206 in RAW264.7-WT, RAW264.7- siScramble or RAW264.7-siSB cells co-cultured with MDA-MB-231 cells. All experiments were performed in triplicate for each condition and repeated at least three times. The data are expressed as the means ± S.E. *P < 0.05, ***P < 0.001
