# Supplementary material for: SerpinB2 deficiency is associated with delayed mammary tumor development and decreased pro-tumorigenic macrophage polarization
Source: BMC Cancer. 2024 Jul 3;24:792. doi: 10.1186/s12885-024-12473-6 (PMC11221169; doi:10.1186/s12885-024-12473-6)
Supplement: Supplementary file 3 — Supplementary Material 3. [file 12885_2024_12473_MOESM3_ESM.docx]

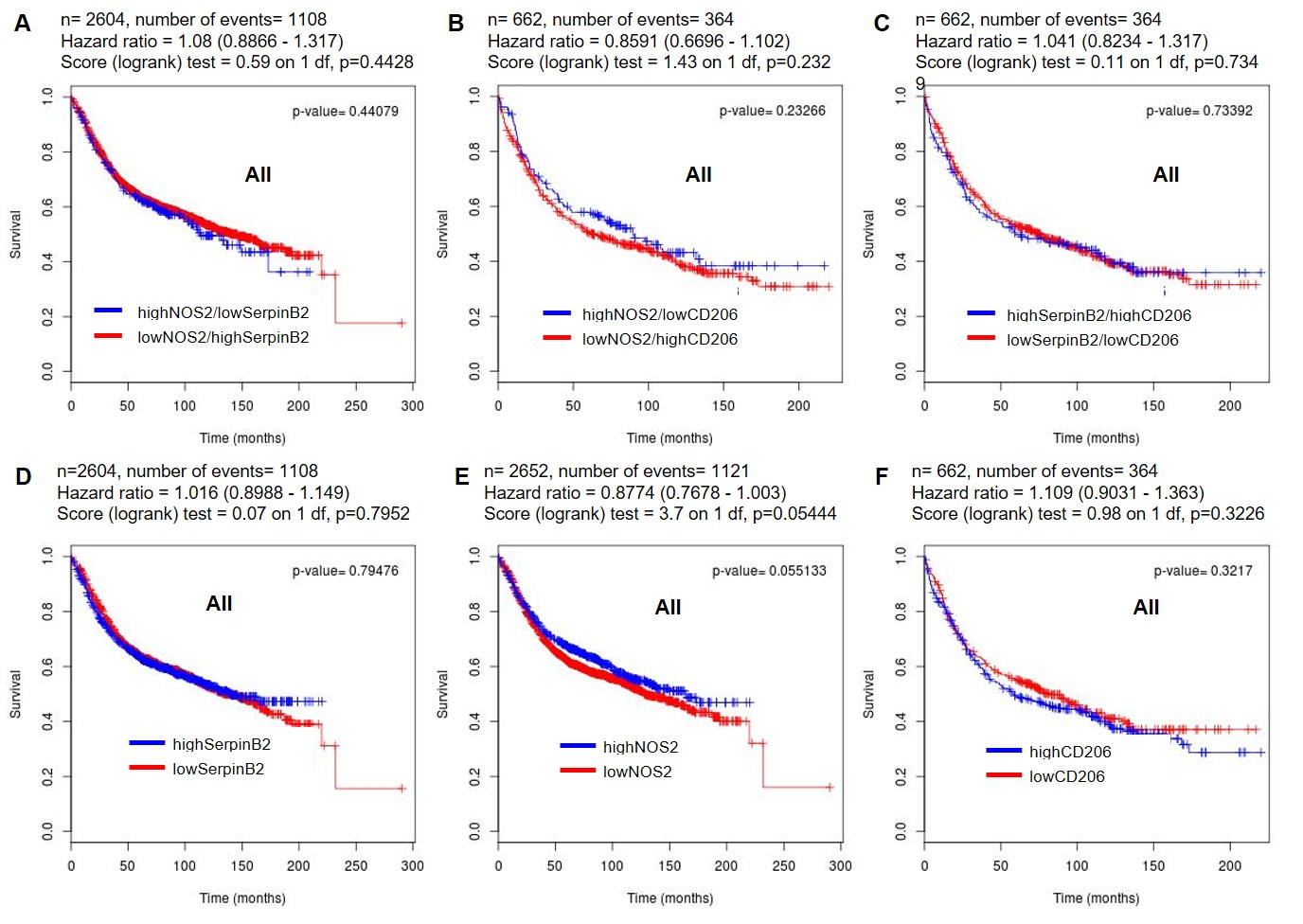


**Supplementary Data 3: Kaplan-Meier plots of breast cancer patient survival based on the combination and individual of SerpinB2, NOS2, and CD206 expression in the BreastMark dataset. A-C** Disease-free survival (DFS) analysis of all breast cancer patients using the combination of SerpinB2/NOS2, SerpinB2/CD206, and NOS2/CD206 groups. **D-F** Disease-free survival (DFS) analysis of all breast cancer patients using the individual expressions of SerpinB2, NOS2, or CD206 alone.
