# Supplementary material for: SerpinB2 deficiency is associated with delayed mammary tumor development and decreased pro-tumorigenic macrophage polarization
Source: BMC Cancer. 2024 Jul 3;24:792. doi: 10.1186/s12885-024-12473-6 (PMC11221169; doi:10.1186/s12885-024-12473-6)
Supplement: Supplementary file 4 — Supplementary Material 4. [file 12885_2024_12473_MOESM4_ESM.docx]

**Table S1. Specific primer sequences for quantitative real-time RT-PCR**

| **Gene** | **Sequence (5′**$\boldsymbol{->}$**3′)** | |
| --- | --- | --- |
| SerpinB2 | Forward | ACTTAATGGGCTTTATCCTTTCC |
|  | Reverse | TGCGTCCTCAATCTCATCG |
| NOS2 | Forward | CAGAGGACCCAGAGACAAGC |
|  | Reverse | TGCTGAAACATTTCCTGTGC |
| IL23α | Forward | CTTGCAAAGGATCCGCCAAG |
|  | Reverse | CTGGGCATCTGTTGGGTCTC |
| CXCL2 | Forward | CTGCCAAGGGTTGACTTCAAGA |
|  | Reverse | GCTTCAGGGTCAAGGCAAACT |
| CXCL13 | Forward | CGGATTCAAGTTACGCCCCC |
|  | Reverse | GCTTGGGGAGTTGAAGACAGA |
| CD206 | Forward | TTCGGTGGACTGTGGACGAGCA |
|  | Reverse | ATAAGCCACCTGCCACTCCGGT |
| CCL17 | Forward | GGCCGAGAGTGCTGCCTGGA |
|  | Reverse | GCCCTGGACAGTCAGAAACACGA |
| IRF4 | Forward | AATTGGTCGAGAGGAGCCAG |
|  | Reverse | CTGTCATGGGGTGGCATCAT |
| CCL8 | Forward | TGCTGCTCATAGCTGTCCCT |
|  | Reverse | TCCATGGGGCACTGGATATTG |
| β-actin | Forward | TTCCTGGGCATGGAGTCCTGTGG |
|  | Reverse | CGCCTAGAAGCATTTGCGGTGG |
