# Supplementary material for: SerpinB2 deficiency is associated with delayed mammary tumor development and decreased pro-tumorigenic macrophage polarization
Source: BMC Cancer. 2024 Jul 3;24:792. doi: 10.1186/s12885-024-12473-6 (PMC11221169; doi:10.1186/s12885-024-12473-6)
Supplement: Supplementary file 5 — Supplementary Material 5. [file 12885_2024_12473_MOESM5_ESM.docx]

**Table S2. GO terms in the biological process (BP) categories in PyMT^SB2−/−^ tumors compared to PyMT^WT^ tumors.**

| **Go term** | **Description** | **Count** | ***P*-value** | | **Genes** | |
| --- | --- | --- | --- | --- | --- | --- |
| **Downregulated** | | | | | | |
| GO:0007155 | Cell adhesion | 49 | | 3.0E-15 | | POSTN, LY9, PCDHGA4, CD96, NEGR1, CNTNAP4, IZUMO1R, SDK1, THY1, CD84, SIGLEC1, BVES, CD36, PGM5, CX3CR1, VCAN, CD226, AOC3, AMTN, ITGAE, COL28A1, ITGAM, EPHB1, KLRA9, AMELX, KLRA8, KLRA4, CD4, KLRA7, AMICA1, KLRA1, COL8A2, MAG, SVEP1, SELL, BMX, COL15A1, NID1, STAB2, ITGA4, EMILIN2, SLAMF1, LAMA2, LYVE1, OMD, CASS4, FREM1, FBLN7, CDH11 |
| GO:0006935 | Chemotaxis | 18 | | 1.4E-08 | | RARRES2, FPR1, CCL8, FGF10, PF4, CXCR3, CCL17, CCL24, CCR7, EAR2, CXCL13, CCR3, CCR2, CXCR6, CX3CR1, PDGFRA, XCL1, XCR1 |
| GO:0006954 | Inflammatory response | 30 | | 4.2E-08 | | RARRES2, FFAR2, GSDMD, FPR1, CCL8, TLR4, PF4, NLRP1B, CXCR3, TLR7, NAIP6, CCL24, PTGIR, CXCR6, REG3G, FAS, CIITA, TLR11, HC, EPHX2, CCL21B, CCL17, CD163, IFI202B, PRKCQ, CCR7, CXCL13, CCR3, CCR2, XCL1 |
| GO:0002376 | Immune system process | 30 | | 4.1E-07 | | FFAR2, FCNA, GSDMD, KLRK1, PTPN22, TLR4, NLRP1B, LY9, SKAP1, TLR7, NAIP6, C1RA, CD4, AMICA1, CFD, TLR11, CRTAM, ADGRE1, HC, EOMES, SLAMF1, PRKCB, MARCH1, CD84, PRKCQ, CD55, TXK, CD79B, MST1R, C1S1 |
| GO:0070374 | Positive regulation of ERK1 and ERK2 cascade | 18 | | 1.1E-05 | | NRP1, PTPN22, CCL21B, FGF10, CCL8, TLR4, SLAMF1, CCL17, CCL24, HCRTR1, CD36, P2RY1, TEK, PDGFRA, ANGPT1, EPOR, XCL1, FGF2 |
| GO:0050731 | Positive regulation of peptidyl-tyrosine phosphorylation | 13 | | 1.4E-05 | | NRP1, FGF10, IGF1, IGF2, TLR4, HGF, ADIPOQ, IL6RA, CD36, PDGFRA, ANGPT1, CD4, EGF |
| GO:0001525 | Angiogenesis | 20 | | 2.1E-05 | | PTPRB, CAV1, NRP1, CCDC80, PDE3B, FGF10, CXCR3, MMP2, EPHB1, NDNF, THY1, LEP, PLXDC1, CCR2, HOXA7, TEK, ANGPT1, EGF, COL8A2, FGF2 |
| GO:0006955 | Immune response | 21 | | 3.9E-05 | | TLR11, C6, MCPT4, CCL8, CCL21B, PF4, COLEC12, TLR7, CCL17, CCL24, MARCH1, CCR7, CD36, H2-EB2, CXCL13, CCR2, H2-OB, TGFBR3, CD4, FAS, XCL1 |
| GO:0030334 | Regulation of cell migration | 10 | | 1.9E-04 | | LAMA2, PLXNC1, PLXNA4, CCR2, TEK, DPYSL3, MMP3, DOCK10, THY1, EPHA3 |
| GO:0045766 | Positive regulation of angiogenesis | 12 | | 3.7E-04 | | CCL24, SEMA5A, HC, CCR3, C6, CX3CR1, TEK, HGF, CXCR3, AQP1, FGF2, PRKCB |
| **Upregulated** | | | | | | |
| GO:0042060 | Wound healing | 6 | | 3.2E-04 | | TRP53, CXCL2, SERPINE1, VANGL2, CELSR1, SDC4 |
| GO:0000122 | Negative regulation of transcription from RNA polymerase II promoter | 14 | | 6.8E-04 | | TRP53, GLIS3, NANOG, C1QBP, VEGFA, NR6A1, TBX20, TSIX, TGIF1, FHL2, TRIB3, LEF1, HES7, ZFP536 |
| GO:0008285 | Negative regulation of cell proliferation | 10 | | 7.3E-04 | | RERG, LIF, TRP53, PTGS2, HNF4A, ADM, TGIF1, HSPA1A, FOSL1, ERDR1 |
| GO:0045766 | Positive regulation of angiogenesis | 6 | | 1.0E-03 | | ADM, VEGFA, SERPINE1, THBS1, VASH2, ANXA3 |
| GO:0045944 | Positive regulation of transcription from RNA polymerase II promoter | 16 | | 1.5E-03 | | TRP53, GLIS3, NANOG, TBX20, NR6A1, NR4A1, LEF1, SOX8, LIF, IL23A, HNF4A, CSRNP1, VEGFA, AIRE, ARNTL2, FOSL1 |
| GO:0001569 | Patterning of blood vessels | 4 | | 2.0E-03 | | VEGFA, TBX20, VANGL2, LEF1 |
| GO:0050796 | Regulation of insulin secretion | 4 | | 3.2E-03 | | SLC16A1, HNF4A, SYT9, NOS2 |
| GO:0001843 | Neural tube closure | 5 | | 3.3E-03 | | ADM, VANGL2, TGIF1, CELSR1, SDC4 |
| GO:0006351 | Transcription, DNA-templated | 22 | | 6.8E-03 | | TRP53, ZCCHC12, GLIS3, NANOG, NR6A1, TBX20, TRIB3, NR4A1, LEF1, CTNND1, FHL2, HES7, SOX8, DAPK3, C1QBP, HNF4A, CSRNP1, AIRE, TGIF1, ZFAT, ARNTL2, ZFP536 |
| GO:0006355 | Regulation of transcription, DNA-templated | 25 | | 7.5E-03 | | GLIS3, TBX20, NR6A1, CTNND1, FHL2, TRIB3, SOX8, MYRFL, ZFAT, ARNTL2, FOSL1, TRP53, ZCCHC12, NANOG, ZFP169, LEF1, NR4A1, HES7, DAPK3, C1QBP, HNF4A, CSRNP1, AIRE, TGIF1, ZFP536 |
