# Supplementary material for: SerpinB2 deficiency is associated with delayed mammary tumor development and decreased pro-tumorigenic macrophage polarization
Source: BMC Cancer. 2024 Jul 3;24:792. doi: 10.1186/s12885-024-12473-6 (PMC11221169; doi:10.1186/s12885-024-12473-6)
Supplement: Supplementary file 6 — Supplementary Material 6. [file 12885_2024_12473_MOESM6_ESM.docx]

**Table S3. Th1 and Th2 cytokines from the peripheral blood serum of 25-week-old PyMT^WT^ and PyMT^SB2−/−^ mice**

| **Th1/Th2 Cytokines** | **PyMT^WT^ (n=5)**  **(pg/ml)** | **PyMT^SB2−/−^ (n=10)**  **(pg/ml)** | ***P*-value** |
| --- | --- | --- | --- |
| IL-2 | 5.11±1.50 | 3.53±0.17 | 0.1929 |
| IL-4 | 3.96±1.14 | 2.67±0.44 | 0.2354 |
| IL-5 | 7.37±1.07 | 6.31±0.98 | 0.4972 |
| IL-6 | 11.49±6.63 | 2.78±0.36 | 0.286 |
| IL-10 | 37.37±5.21 | 31.59±4.47 | 0.4265 |
| IL-12(p70) | 71.99±16.47 | 35.43±9.96 | 0.0621 |
| IL-13 | 29.04±5.35 | 19.37±2.90 | 0.2562 |
| GM-CSF | 29.08±10.35 | 25.48±7.46 | 0.7776 |
| INF-γ | 9.01±2.33 | 3.76±0.94 | 0.0283 |
| TNF-α | 40.37±10.38 | 19.32±4.12 | 0.0442 |

The data were represented as the means ± S.E. from the blood serum of 7 mice per group. Statistical analysis by t-test
